# Associations of thyroid hormones with chronic diarrhea and constipation in euthyroid individuals

**DOI:** 10.3389/fendo.2024.1395743

**Published:** 2024-10-17

**Authors:** Weiwei Zeng, Yanjun Wang, Luofang Zhang

**Affiliations:** ^1^ Department of Hepatology, The First Affiliated Hospital of Fujian Medical University, Fuzhou, Fujian, China; ^2^ Department of Gastroenterology, Army Medical Center of PLA, Daping Hospital, Army Medical University, Chongqing, China

**Keywords:** chronic constipation, chronic diarrhea, number of bowel movements, thyroid hormones, NHANES

## Abstract

**Background:**

Abnormalities in thyroid function affect bowel health. However, the relationships between thyroid hormone concentrations and the risk of developing chronic diarrhea and constipation remain unclear. Thus, the aim of this study was to investigate the relationships between thyroid hormone concentrations and the risk of developing chronic diarrhea and constipation in euthyroid US adults.

**Methods:**

The data for this population-based study were taken from the National Health and Nutrition Examination Survey (NHANES) 2007–2010 datasets. The relationships between thyroid hormone concentrations and the risk of developing chronic diarrhea and constipation were examined via multivariate regression. Smoothed curve fitting and threshold effects analysis were used to test for nonlinear relationships and inflection points.

**Results:**

This study involved 4999 participants ranging in age from 20 to 80 years. Multivariate logistic regression analysis revealed a significant positive correlation between FT3 concentrations and the risk of developing chronic diarrhea [1.37 (1.00, 1.88), *P*=0.049]. Multivariate linear regression analysis revealed a significant positive correlation between FT3 concentrations and the number of bowel movements [0.84 (0.39, 1.28), *P*<0.001]. Using smoothed curve fitting and the two-stage regression model, we found a nonlinear relationship between FT4 concentrations and chronic diarrhea, with a breakpoint of 0.79 ng/dl.

**Conclusions:**

There were associations between thyroid hormone concentrations and abnormal bowel habits, particularly between FT3 concentrations and the risk of developing chronic diarrhea. A higher FT3 level was associated with an increased risk of developing chronic diarrhea and more frequent bowel movements. To validate our results, further large-scale prospective studies are needed.

## Introduction

Functional constipation, functional diarrhea and irritable bowel syndrome are common functional gastrointestinal diseases (FGIDs) that seriously interfere with a patient’s normal life and require a large amount of medical resources. Two-thirds of these patients have chronic, fluctuating symptoms (1). The global prevalence rates of functional constipation and diarrhea are estimated to be 11% and 5%, respectively (1). The prevalence of irritable bowel syndrome was reported to be 9.2% in a systematic review (2). These diseases are characterized by a lack of pathologic changes and biochemical abnormalities that explain symptoms. The current Rome criteria define FGIDs as disorders of the gut−brain interaction (3). Abnormal gastrointestinal motility and visceral hypersensitivity are considered to contribute to the pathogenesis of FGIDs (1, 4). In recent years, the involvement of the neuroendocrine axis in visceral hypersensitivity and gastrointestinal motility disorders has attracted increasing attention (5).

The hypothalamic−pituitary−thyroid (HPT) axis is an essential branch of the neuroendocrine network. Thyroid-stimulating hormone (TSH) is synthesized and secreted in response to hypothalamic thyrotropin-releasing hormone (TRH). TSH operates at the thyroid to activate thyroid hormone production. To maintain physiological levels of the primary hormones of the HPT axis, triiodothyronine (T3) and thyroxine (T4) regulate the production of TRH and TSH via negative feedback. The active hormone T3 is produced by the thyroid gland at a lower level than T4. To be physiologically active, T4 must be converted into T3 in peripheral tissues since only T3 interacts with the thyroid hormone receptor (TR) (6, 7).

The HPT axis is involved in visceral sensation and gastrointestinal motility, such as promoting intestinal peristalsis (8). For example, hyperthyroidism is associated with an increased number of bowel movements (9). The mouth-to-cecum transit time was inversely linked with T3 levels in hyperthyroid patients (10). Propylthiouracil therapy for hyperthyroidism can alleviate diarrhea and restore the mouth-to-caecum transit time (11). Moreover, T3 concentrations were found to be positively correlated with gastrin levels and negatively correlated with acid output (11). Previous studies have reported that thyroid hormone deficiency results in decreased colonic motility, and reduced basic electrical rhythms by altering hormone receptors or modulating neuromuscular contractions in the gastrointestinal tract (12). Hypothyroidism causes glycosaminoglycans to accumulate in the interstitial tissues or smooth muscle of the gastrointestinal tract, which delays bowels transit (13).

The associations of thyroid function with constipation and diarrhea, however, have not been studied in large, population-based studies. On the basis of data from the National Health and Nutrition Examination Survey (NHANES), we conducted a cross-sectional study to investigate the relationships between thyroid hormone concentrations and bowel habits in euthyroid individuals.

## Methods

### Study population

The study data, including demographic information, laboratory examination data, questionnaires, and disease history data, were derived from NHANES, a continuous survey focused on the U.S. civilian population that uses a complex, multistage, and probabilistic sampling design to assess the health and nutritional status of adults and children.

All NHANES data are publicly available at https://www.cdc.gov/nchs/nhanes/. Our study was based on two NHANES survey cycles from 2007 to 2010. The data for the 2007-2008 cycle comes from the following website: https://wwwn.cdc.gov/nchs/nhanes/continuousnhanes/default.aspx?BeginYear=2007; The data for the 2009-2010 cycle comes from the following website: https://wwwn.cdc.gov/nchs/nhanes/continuousnhanes/default.aspx?BeginYear=2009.

**Figure 1:**
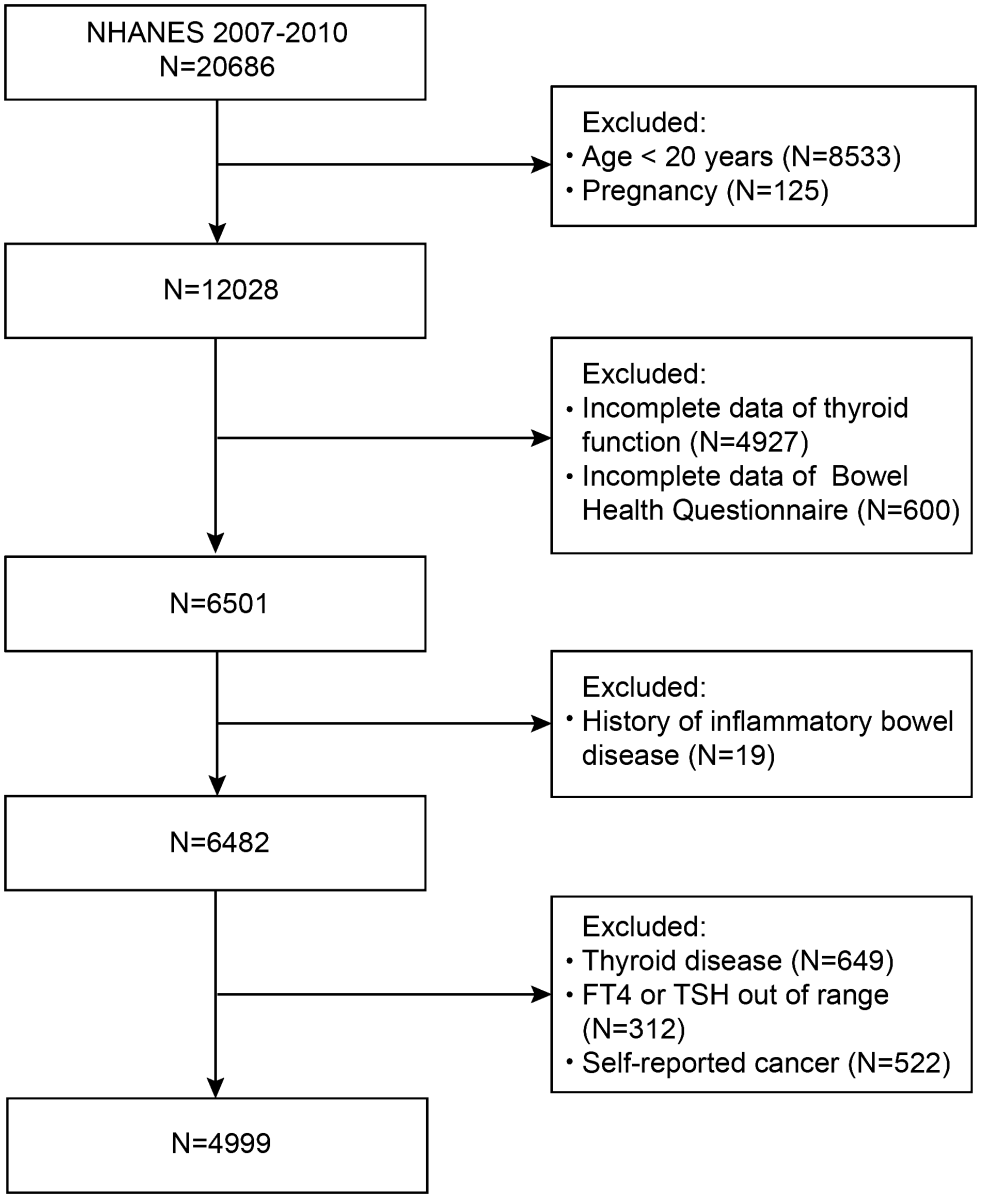
Flowchart of the sample selection.

First, in this study, we did not include people under 20 years of age (n=8533) or those who were pregnant (n=125). Participants who lacked thyroid profile data (n=4927) or bowel health questionnaire data (n=600) were also excluded from the study. A further 668 participants with inflammatory bowel disease (n=19) or thyroid disease (n=649) were excluded. Moreover, those with FT4 or TSH levels outside the normal limits (n=312) and those with cancer (n=522) were also disqualified. Eventually, 4999 subjects were enrolled in this research (**Figure 1**). All the subjects provided written informed consent. The procedures of the NHANES program were approved by the National Center for Health Statistics (NCHS) Ethics Review Board.

### Bowel health questionnaire

The participants were classified as having chronic diarrhea or chronic constipation according to their answers to the bowel health questionnaire. Participants were shown a card featuring colored illustrations and descriptions of the seven Bristol Stool Form Scale (BSFS) types and asked to tell interviewers “the number that corresponds with your usual or most common stool type.” Participants with a typical BSFS type 1 (separate hard lumps, like nuts) or type 2 (sausage-like, but lumpy) stool type were categorized as having chronic constipation, whereas those with a typical BSFS type 6 (fluffy pieces with ragged edges, a mushy stool) or type 7 (watery, no solid pieces) stool type were considered to have chronic diarrhea (14, 15). The remaining individuals are thought to have normal bowel habits. The question, “How often do you have bowel movements?” was also posed to the participants. The responses that were recorded as times per day were converted to times per week so that all the responses were presented as times per week.

### Assessment of thyroid function

The chemiluminescence technique was used to determine the levels of thyroid hormones. Total triiodothyronine (TT3), free triiodothyronine (FT3), and total thyroxine (TT4) concentrations were measured via a competitive binding immunoenzymatic assay. Free thyroxine (FT4) concentrations were measured via the Access FT4 assay, a two-step enzyme immunoassay. TSH concentrations were measured via a two-site immunoenzymatic assay (“sandwich”). According to previous research, the normal range of TSH is 0.34–5.60 mIU/L, and that of FT4 is 0.6–1.6 ng/dL (16). Participants were classified as euthyroid if their serum TSH and FT4 levels were within the normal limits (16, 17).

### Covariates

Covariates in the study included age, gender, race (Mexican American, Non-Hispanic white, Non-Hispanic black, other Hispanic, and other races), educational level (less than high school, high school or equivalent and more than high school), marital status (married/cohabiting with partner and others), Patient Health Questionnaire (PHQ-9) score, energy intake, total calcium, smoking status (smoked ≥ 100 cigarettes in life, yes or no), frequency of alcohol consumption (<12 drinks per year or ≥12 drinks per year), hypertension, diabetes, body mass index (BMI) (18), and physical activity.

### Statistical analysis

Continuous variables are presented as the means ± standard deviations (SDs). Categorical variables are expressed as proportions. Comparisons among groups divided by bowel habits were evaluated by a weighted one-way ANOVA (for continuous variables) or a weighted chi-square test (for categorical variables). The relationships between thyroid function and abnormal bowel habits were examined via multivariate logistic regression. The relationships between thyroid function and the number of bowel movements were examined via multivariate linear regression. For multivariate tests, the following three models were employed. Model 1 was not adjusted for any variables. Model 2 was adjusted for age, gender, and race. Model 3 was adjusted for age, gender, race, educational level, marital status, BMI, alcohol use, smoking status, total calcium, energy intake, PHQ-9 score, hypertension status, diabetes status, and physical activity. Stratified subgroups were analyzed for gender, age, and BMI (<25 kg/m^2^, 25–30 kg/m^2^, and ≥30 kg/m^2^) via a multivariate regression model. Moreover, the nonlinear relationships of thyroid hormone (FT3, FT4) levels with the risk of developing chronic diarrhea and the number of bowel movements were investigated through the application of smoothed curve fitting. Threshold effect analysis was used to investigate the inflection points of thyroid hormone (FT3, FT4) levels with the risk of developing chronic diarrhea and the number of bowel movements. EmpowerStats (version 2.0) and R (version 3.4.3) software were used to analyze the statistics. *P* values < 0.05 were considered to indicate statistical significance.

## Results

### Baseline characteristics of the participants

In this study, 4999 participants were categorized into groups on the basis of normal bowel habits, chronic constipation, or chronic diarrhea status. A total of 7.32% of the population exhibited chronic constipation, and 7.72% exhibited chronic diarrhea. The participants’ average age was 47.63 ± 17.07 years. Among these participants, 53.69% were male and 46.31% were female. Abnormal bowel habits were more prevalent in females than in males (*P*<0.001). Compared with participants with chronic constipation and those with chronic diarrhea, those with normal bowel habits comprised a greater proportion of the population with higher educational levels and a lower PHQ-9 score. Compared with those in the normal group, participants in the chronic constipation group tended to be younger and have a lower BMI (*P*<0.05). In the chronic constipation group, FT4 levels and weekly bowel movements were greatly decreased (all *P*<0.05). Among the three groups, participants with chronic diarrhea tended to have more frequent bowel movements. They also had lower rates of moderate recreational activities and higher rates of smoking, hypertension, and diabetes (all *P*<0.05). No discernible differences in terms of race; vigorous/moderate work activities; or hormone levels of FT3, TT3, TT4, and TSH were detected among the three groups (**Table 1**).

**Table 1:** Baseline characteristics of participants based on bowel health.

Characteristics	Normal bowel habits (n=4247)	Chronic constipation (n=366)	Chronic diarrhea (n=386)	*P* value
Age (years)	44.20 ± 15.54	42.49 ± 16.08 ^a^	46.91 ± 15.50 ^a^	<0.001
Gender (%)				<0.001
Male	55.76	33.74 ^a^	48.29 ^a^	
Female	44.24	66.26	51.71	
Race/Ethnicity (%)				0.119
Mexican American	9.45	8.56	11.90	
Other Hispanic	5.31	7.50	6.69	
Non-Hispanic White	68.09	64.28	64.83	
Non-Hispanic Black	10.79	13.85	12.31	
Other Race	6.36	5.81	4.27	
Education Level (%)				<0.001
Less than high school	19.55	22.29 ^a^	26.79 ^a^	
High school	23.99	31.92	21.92	
More than high school	56.46	45.79	51.30	
Marital Status (%)				0.015
Married/Living with partner	64.23	56.73 ^a^	64.73	
Others	35.77	43.27	35.27	
BMI (kg/m^2^)	28.46 ± 6.38	27.74 ± 6.94 ^a^	29.18 ± 7.59	0.014
Alcohol use (%)				<0.001
<12 drinks per year	21.96	31.84 ^a^	25.78	
≥12 drinks per year	78.04	68.16	74.22	
Smoked at least 100 cigarettes (%)				0.003
YES	46.36	42.63	54.79 ^a^	
NO	53.64	57.37	45.21	
Vigorous work activities (%)				0.380
YES	23.80	21.34	21.45	
NO	76.20	78.66	78.55	
Moderate work activities (%)				0.691
YES	43.82	41.74	42.60	
NO	56.18	58.26	57.40	
Vigorous recreational activities (%)				0.049
YES	28.03	22.78 ^a^	24.66	
NO	71.97	77.22	75.34	
Moderate recreational activities (%)				0.012
YES	44.74	40.89	37.10 ^a^	
NO	55.26	59.11	62.90	
Hypertension (%)				0.002
YES	26.16	24.78	34.74 ^a^	
NO	73.84	75.22	65.26	
Diabetes (%)				<0.001
YES	6.62	6.18	12.64 ^a^	
NO	93.38	93.82	87.36	
PHQ-9 score	2.78 ± 3.65	4.05 ± 4.73	4.10 ± 4.88	<0.001
Energy intake (kcal/d)	2265.59 ± 1080.15	2041.64 ± 906.50	2160.66 ± 962.73	<0.001
Total calcium (mg/dL)	9.43 ± 0.36	9.41 ± 0.36	9.42 ± 0.38	0.513
Bowel movements per week (times)	9.27 ± 4.94	8.22 ± 5.45 ^a^	12.22 ± 6.96 ^a^	<0.001
Thyroid hormone				
FT3 (pg/mL)	3.22 ± 0.37	3.19 ± 0.38	3.23 ± 0.46	0.298
FT4 (ng/dL)	0.78 ± 0.12	0.77 ± 0.11 ^a^	0.79 ± 0.13	0.033
TSH (mIU/L)	1.82 ± 0.99	1.75 ± 0.92	1.78 ± 0.96	0.378
TT3 (ng/dL)	114.58 ± 22.15	115.27 ± 22.68	115.62 ± 26.55	0.636
TT4 (ug/dL)	7.75 ± 1.48	7.78 ± 1.45	7.87 ± 1.54	0.333

Mean ± SD for continuous variables. % for categorical variables.

a Compared with normal bowel habits group, P<0.05.

BMI, body mass index; PHQ-9, Patient Health Questionnaire; FT3, free triiodothyronine; FT4, free thyroxine; TSH, thyroid stimulating hormone; TT3, total triiodothyronine; TT4, total thyroxine.

### Correlations of thyroid hormone levels with abnormal bowel habits and the number of bowel movements per week

The results of logistic regression analysis in Model 3 revealed negative but nonsignificant relationships between thyroid hormone (FT4 and TSH) concentrations and the risk of developing chronic constipation (**Table 2**). However, in Model 2, FT3 concentrations were significantly positively related to chronic constipation status in the 20–40-year-old population [1.59 (1.01, 2.49), *P*=0.045]. After comparing gender differences in thyroid function in different age groups, we found that men had significantly higher FT3 concentrations than women (*P*<0.001) (**Supplementary Table 1**). Further analyzing the relationships between thyroid function and chronic constipation in different age groups, we found a significant negative correlation between FT3 concentration and chronic constipation in the male 60–80-year-old population (*P*<0.05) (**Supplementary Table 2**).

**Table 2:** The associations of thyroid hormone with chronic constipation.

Exposure	OR (95% CI), *P* value		
	Model 1 (Non-adjusted)	Model 2 (Adjust I)	Model 3 (Adjust II)
Total			
FT3 (pg/mL)	0.90 (0.68, 1.20)	1.05 (0.77, 1.42)	1.05 (0.76, 1.46)
FT4 (ng/dL)	0.67 (0.27, 1.63)	0.85 (0.34, 2.12)	0.82 (0.32, 2.10)
TSH (mIU/L)	0.93 (0.83, 1.04)	0.94 (0.84, 1.06)	0.96 (0.86, 1.08)
TT3 (ng/dL)	1.00 (1.00, 1.01)	1.00 (1.00, 1.01)	1.00 (1.00, 1.01)
TT4 (ug/dL)	1.03 (0.96, 1.10)	1.00 (0.93, 1.07)	1.00 (0.93, 1.07)
Gender			
Male			
FT3 (pg/mL)	1.13 (0.72, 1.78)	0.95 (0.56, 1.59)	0.90 (0.52, 1.56)
FT4 (ng/dL)	0.91 (0.22, 3.75)	1.01 (0.24, 4.27)	0.89 (0.20, 3.99)
TSH (mIU/L)	0.99 (0.82, 1.18)	1.01 (0.84, 1.21)	0.98 (0.81, 1.19)
TT3 (ng/dL)	1.01 (1.00, 1.01)	1.00 (1.00, 1.01)	1.01 (1.00, 1.01)
TT4 (ug/dL)	1.03 (0.92, 1.16)	1.04 (0.92, 1.17)	1.02 (0.90, 1.15)
Female			
FT3 (pg/mL)	1.18 (0.83, 1.69)	1.10 (0.76, 1.60)	1.18 (0.78, 1.80)
FT4 (ng/dL)	0.65 (0.20, 2.06)	0.75 (0.23, 2.45)	0.74 (0.22, 2.48)
TSH (mIU/L)	0.88 (0.76, 1.02)	0.91 (0.78, 1.05)	0.94 (0.81, 1.09)
TT3 (ng/dL)	1.00 (1.00, 1.01)	1.00 (1.00, 1.01)	1.00 (1.00, 1.01)
TT4 (ug/dL)	0.98 (0.89, 1.06)	0.98 (0.89, 1.07)	0.99 (0.90, 1.08)
Age			
20-40 years old			
FT3 (pg/mL)	1.06 (0.69, 1.64)	1.59 (1.01, 2.49) *	1.57 (0.98, 2.49)
FT4 (ng/dL)	2.66 (0.65, 10.89)	4.15 (0.97, 17.66)	3.95 (0.90, 17.27)
TSH (mIU/L)	0.98 (0.82, 1.19)	1.00 (0.83, 1.21)	1.01 (0.83, 1.23)
TT3 (ng/dL)	1.01 (1.00, 1.01)	1.01 (1.00, 1.01)	1.01 (1.00, 1.01)
TT4 (ug/dL)	1.04 (0.93, 1.16)	0.99 (0.88, 1.11)	0.99 (0.88, 1.10)
40-60 years old			
FT3 (pg/mL)	0.52 (0.30, 0.89) *	0.67 (0.39, 1.17)	0.62 (0.34, 1.12)
FT4 (ng/dL)	0.27 (0.06, 1.33)	0.32 (0.07, 1.58)	0.24 (0.04, 1.26)
TSH (mIU/L)	0.83 (0.68, 1.01)	0.83 (0.68, 1.02)	0.83 (0.68, 1.02)
TT3 (ng/dL)	1.00 (0.99, 1.01)	1.00 (0.99, 1.01)	1.00 (0.99, 1.01)
TT4 (ug/dL)	1.04 (0.92, 1.16)	1.01 (0.90, 1.14)	1.01 (0.89, 1.14)
60-80 years old			
FT3 (pg/mL)	0.75 (0.38, 1.49)	0.88 (0.44, 1.78)	0.75 (0.34, 1.65)
FT4 (ng/dL)	0.35 (0.06, 2.02)	0.27 (0.04, 1.64)	0.37 (0.06, 2.37)
TSH (mIU/L)	1.04 (0.85, 1.27)	1.00 (0.81, 1.23)	1.00 (0.80, 1.25)
TT3 (ng/dL)	1.00 (0.99, 1.01)	1.00 (0.99, 1.01)	1.00 (0.99, 1.01)
TT4 (ug/dL)	1.01 (0.87, 1.16)	0.98 (0.85, 1.14)	1.00 (0.86, 1.17)
BMI			
<25 kg/m^2^			
FT3 (pg/mL)	0.72 (0.45, 1.18)	0.91 (0.52, 1.60)	0.89 (0.50, 1.59)
FT4 (ng/dL)	1.62 (0.39, 6.65)	2.20 (0.50, 9.70)	1.89 (0.40, 8.78)
TSH (mIU/L)	0.99 (0.82, 1.18)	1.00 (0.83, 1.21)	1.00 (0.83, 1.22)
TT3 (ng/dL)	1.00 (0.99, 1.01)	1.00 (0.99, 1.01)	1.00 (0.99, 1.01)
TT4 (ug/dL)	1.06 (0.95, 1.18)	1.03 (0.92, 1.16)	1.01 (0.90, 1.14)
25-30 kg/m^2^			
FT3 (pg/mL)	1.07 (0.68, 1.68)	1.34 (0.85, 2.12)	1.32 (0.76, 2.28)
FT4 (ng/dL)	0.29 (0.06, 1.45)	0.41 (0.08, 2.16)	0.39 (0.07, 2.15)
TSH (mIU/L)	0.86 (0.70, 1.06)	0.86 (0.70, 1.07)	0.87 (0.70, 1.09)
TT3 (ng/dL)	1.01 (1.00, 1.01)	1.01 (1.00, 1.01)	1.01 (1.00, 1.01)
TT4 (ug/dL)	1.04 (0.92, 1.18)	1.02 (0.90, 1.16)	1.02 (0.90, 1.16)
≥30 kg/m^2^			
FT3 (pg/mL)	1.03 (0.61, 1.74)	1.04 (0.59, 1.85)	0.99 (0.55, 1.79)
FT4 (ng/dL)	0.52 (0.10, 2.69)	0.65 (0.12, 3.43)	0.59 (0.11, 3.29)
TSH (mIU/L)	0.99 (0.81, 1.20)	1.02 (0.83, 1.24)	1.01 (0.82, 1.23)
TT3 (ng/dL)	1.00 (1.00, 1.01)	1.00 (0.99, 1.01)	1.00 (0.99, 1.01)
TT4 (ug/dL)	1.00 (0.88, 1.14)	0.97 (0.85, 1.11)	0.94 (0.82, 1.08)

*P<0.05; **P<0.01; ***P<0.001.

FT3, free triiodothyronine; FT4, free thyroxine; TSH, thyroid stimulating hormone; TT3, total triiodothyronine; TT4, total thyroxine; BMI, body mass index.

Model 2: Adjusted for age, gender and race. Model 3: Adjusted for age, gender, race, education, marriages, body mass index, alcohol use, smoking status, total calcium, energy intake, Patient Health Questionnaire score, hypertension, diabetes and physical activities. All the models are not adjusted for the variable itself in each stratification.

Moreover, a positive correlation between FT3 concentrations and the risk of developing chronic diarrhea was observed in Model 3 [1.37 (1.00, 1.88), *P*=0.049]. When subgroups were analyzed by gender, the results suggested that the positive associations of FT3 [1.77 (1.12, 2.80), *P*=0.015] and TT3 [1.01 (1.00, 1.02), *P*=0.003] concentrations with the risk of developing chronic diarrhea were significantly positive in male participants, whereas higher FT4 concentrations were markedly positively associated with the risk of developing chronic diarrhea in women [3.33 (1.00, 11.08), *P*=0.049]. When comparing the relationships between thyroid function and chronic diarrhea in different age groups, we found that FT3 concentrations were positively associated with chronic diarrhea in the male 20–40-year-old population(all *P*<0.05). For women, FT4 concentrations were significantly positively associated with chronic diarrhea in the 40–60-year-old population (all *P*<0.05) (**Supplementary Table 3**). In the 60–80-year-old population, the relationships between the above hormones and diarrhea were not significant. According to the subgroup analyses stratified by BMI, FT3 concentrations were significantly positively correlated with the risk of developing chronic diarrhea in the 25-30 kg/m^2^ group [1.72 (1.10, 2.70), *P*=0.019] in Model 2 (**Table 3**).

**Table 3:** The associations of thyroid hormone with chronic diarrhea.

Exposure	OR (95% CI), *P* value		
	Model 1 (Non-adjusted)	Model 2 (Adjust I)	Model 3 (Adjust II)
Total			
FT3 (pg/mL)	1.05 (0.80, 1.37)	1.47 (1.11, 1.96) **	1.37 (1.00, 1.88) *
FT4 (ng/dL)	1.64 (0.71, 3.81)	1.60 (0.69, 3.75)	1.47 (0.61, 3.54)
TSH (mIU/L)	1.03 (0.93, 1.14)	1.00 (0.89, 1.11)	0.98 (0.88, 1.09)
TT3 (ng/dL)	1.00 (1.00, 1.01)	1.00 (1.00, 1.01)	1.00 (1.00, 1.01)
TT4 (ug/dL)	1.06 (0.99, 1.13)	1.02 (0.96, 1.10)	1.01 (0.94, 1.09)
Gender			
Male			
FT3 (pg/mL)	1.09 (0.73, 1.62)	1.59 (1.02, 2.48) *	1.77 (1.12, 2.80) *
FT4 (ng/dL)	0.71 (0.20, 2.48)	0.70 (0.20, 2.48)	0.52 (0.14, 1.96)
TSH (mIU/L)	1.02 (0.88, 1.19)	0.98 (0.84, 1.15)	0.95 (0.81, 1.13)
TT3 (ng/dL)	1.01 (1.00, 1.01)	1.01 (1.00, 1.02) *	1.01 (1.00, 1.02) **
TT4 (ug/dL)	1.06 (0.96, 1.17)	1.04 (0.93, 1.15)	1.04 (0.93, 1.16)
Female			
FT3 (pg/mL)	1.26 (0.87, 1.82)	1.42 (0.98, 2.07)	1.07 (0.69, 1.67)
FT4 (ng/dL)	3.88 (1.22, 12.29) *	3.37 (1.05, 10.81) *	3.33 (1.00, 11.08) *
TSH (mIU/L)	1.03 (0.89, 1.18)	1.01 (0.87, 1.17)	1.00 (0.86, 1.16)
TT3 (ng/dL)	1.00 (0.99, 1.01)	1.00 (0.99, 1.01)	1.00 (0.99, 1.00)
TT4 (ug/dL)	1.03 (0.94, 1.12)	1.01 (0.92, 1.10)	0.99 (0.90, 1.08)
Age			
20-40 years old			
FT3 (pg/mL)	1.33 (0.81, 2.18)	1.67 (1.00, 2.80)	1.48 (0.87, 2.51)
FT4 (ng/dL)	0.77 (0.14, 4.30)	0.93 (0.16, 5.42)	0.78 (0.13, 4.70)
TSH (mIU/L)	1.12 (0.92, 1.38)	1.11 (0.90, 1.36)	1.04 (0.84, 1.29)
TT3 (ng/dL)	1.00 (0.99, 1.01)	1.00 (0.99, 1.01)	1.00 (0.99, 1.01)
TT4 (ug/dL)	1.04 (0.92, 1.18)	1.01 (0.88, 1.15)	0.99 (0.86, 1.13)
40-60 years old			
FT3 (pg/mL)	1.50 (0.97, 2.30)	1.54 (0.99, 2.39)	1.31 (0.75, 2.27)
FT4 (ng/dL)	2.93 (0.69, 12.41)	3.08 (0.72, 13.15)	3.87 (0.85, 17.62)
TSH (mIU/L)	0.96 (0.80, 1.16)	0.98 (0.81, 1.17)	0.96 (0.80, 1.17)
TT3 (ng/dL)	1.00 (1.00, 1.01)	1.00 (1.00, 1.01)	1.00 (0.99, 1.01)
TT4 (ug/dL)	1.06 (0.95, 1.19)	1.04 (0.93, 1.17)	1.04 (0.92, 1.17)
60-80 years old			
FT3 (pg/mL)	1.22 (0.72, 2.08)	1.23 (0.72, 2.12)	1.37 (0.77, 2.45)
FT4 (ng/dL)	1.38 (0.38, 5.02)	1.52 (0.41, 5.64)	1.13 (0.29, 4.39)
TSH (mIU/L)	0.94 (0.79, 1.11)	0.95 (0.80, 1.13)	0.95 (0.79, 1.13)
TT3 (ng/dL)	1.01 (1.00, 1.01)	1.00 (1.00, 1.01)	1.01 (1.00, 1.02)
TT4 (ug/dL)	1.04 (0.93, 1.16)	1.02 (0.91, 1.14)	1.01 (0.90, 1.15)
BMI			
<25 kg/m^2^			
FT3 (pg/mL)	0.79 (0.45, 1.38)	1.39 (0.74, 2.61)	1.20 (0.63, 2.29)
FT4 (ng/dL)	0.33 (0.06, 1.93)	0.32 (0.06, 1.90)	0.20 (0.03, 1.26)
TSH (mIU/L)	1.05 (0.85, 1.28)	0.98 (0.79, 1.21)	0.98 (0.79, 1.21)
TT3 (ng/dL)	1.00 (0.99, 1.01)	1.00 (0.99, 1.01)	1.00 (0.99, 1.01)
TT4 (ug/dL)	0.96 (0.84, 1.10)	0.94 (0.82, 1.09)	0.93 (0.80, 1.07)
25-30 kg/m^2^			
FT3 (pg/mL)	1.40 (0.92, 2.13)	1.72 (1.10, 2.70) *	1.47 (0.84, 2.58)
FT4 (ng/dL)	2.99 (0.68, 13.18)	2.56 (0.56, 11.79)	2.32 (0.49, 10.93)
TSH (mIU/L)	1.11 (0.92, 1.33)	1.07 (0.89, 1.30)	1.09 (0.89, 1.32)
TT3 (ng/dL)	1.01 (1.00, 1.01)	1.01 (1.00, 1.01) *	1.00 (1.00, 1.01)
TT4 (ug/dL)	1.07 (0.95, 1.21)	1.04 (0.91, 1.18)	1.04 (0.92, 1.19)
≥30 kg/m^2^			
FT3 (pg/mL)	0.99 (0.64, 1.53)	1.33 (0.83, 2.14)	1.36 (0.83, 2.21)
FT4 (ng/dL)	3.25 (0.89, 11.80)	3.33 (0.90, 12.35)	3.68 (0.94, 14.44)
TSH (mIU/L)	0.91 (0.77, 1.08)	0.91 (0.77, 1.08)	0.90 (0.76, 1.08)
TT3 (ng/dL)	1.00 (0.99, 1.01)	1.00 (0.99, 1.01)	1.00 (0.99, 1.01)
TT4 (ug/dL)	1.10 (1.00, 1.22)	1.06 (0.96, 1.18)	1.05 (0.95, 1.17)

*P<0.05; **P<0.01; ***P<0.001.

FT3, free triiodothyronine; FT4, free thyroxine; TSH, thyroid stimulating hormone; TT3, total triiodothyronine; TT4, total thyroxine; BMI, body mass index.

Model 2: Adjusted for age, gender and race. Model 3: Adjusted for age, gender, race, education, marriages, body mass index, alcohol use, smoking status, total calcium, energy intake, Patient Health Questionnaire score, hypertension, diabetes and physical activities. All the models are not adjusted for the variable itself in each stratification.

According to linear regression analysis, significantly positive correlations were identified between FT3 concentrations and the number of bowel movements [0.84 (0.39, 1.28), *P*<0.001]. When a subgroup analysis was performed on the basis of gender, FT3 concentrations were substantially positively correlated with the number of bowel movements in both the male and female subgroups in the three models (all *P*<0.05). FT4 concentrations were significantly negatively correlated with the number of bowel movements in the female group in Model 2 (*P*<0.05). In subgroup analyses stratified by age, within the 40–60-year age group, associations between FT3 concentrations and the number of bowel movements were positive according to the three models (all *P*<0.05). Within the 60–80-year age group, FT3 concentrations were positively correlated with the number of bowel movements (all *P*<0.05). In subgroup analyses stratified by BMI, within the BMI<25 kg/m^2^, 25–30 kg/m^2^, and ≥30 kg/m^2^ groups, FT3 concentrations were positively correlated with the number of bowel movements in Model 1 and Model 2 (all *P*<0.05). In the BMI<25 kg/m^2^ group, FT4 concentrations were negatively correlated with the number of bowel movements in three models (all *P*<0.05) (**Table 4**).

**Table 4:** The associations of thyroid hormone with bowel movements per week.

Exposure	β (95% CI), *P* value		
	Model 1 (Non-adjusted)	Model 2 (Adjust I)	Model 3 (Adjust II)
Total			
FT3 (pg/mL)	1.55 (1.16, 1.93) ***	1.00 (0.58, 1.43) ***	0.84 (0.39, 1.28) ***
FT4 (ng/dL)	-0.85 (-2.08, 0.38)	-0.96 (-2.18, 0.26)	-0.83 (-2.07, 0.41)
TSH (mIU/L)	-0.03 (-0.18, 0.12)	0.05 (-0.11, 0.20)	0.01 (-0.15, 0.16)
TT3 (ng/dL)	0.01 (0.00, 0.01) *	0.00 (-0.00, 0.01)	0.00 (-0.01, 0.01)
TT4 (ug/dL)	-0.10 (-0.20, 0.00)	-0.07 (-0.17, 0.02)	-0.09 (-0.19, 0.01)
Gender			
Male			
FT3 (pg/mL)	1.27 (0.75, 1.79) ***	0.81 (0.24, 1.39) **	0.73 (0.14, 1.32) *
FT4 (ng/dL)	-0.62 (-2.22, 0.98)	-0.39 (-1.99, 1.21)	-0.22 (-1.85, 1.41)
TSH (mIU/L)	-0.00 (-0.21, 0.20)	0.11 (-0.09, 0.32)	0.08 (-0.13, 0.29)
TT3 (ng/dL)	0.01 (0.00, 0.02) *	0.00 (-0.01, 0.01)	0.00 (-0.01, 0.01)
TT4 (ug/dL)	-0.05 (-0.19, 0.08)	-0.07 (-0.20, 0.07)	-0.06 (-0.20, 0.07)
Female			
FT3 (pg/mL)	0.92 (0.31, 1.53) **	1.06 (0.42, 1.69) **	0.87 (0.19, 1.56) *
FT4 (ng/dL)	-1.62 (-3.50, 0.26)	-2.03 (-3.91, -0.14) *	-1.88 (-3.79, 0.03)
TSH (mIU/L)	-0.03 (-0.25, 0.19)	-0.04 (-0.26, 0.19)	-0.07 (-0.30, 0.15)
TT3 (ng/dL)	0.00 (-0.01, 0.01)	0.00 (-0.01, 0.01)	0.00 (-0.01, 0.01)
TT4 (ug/dL)	-0.00 (-0.15, 0.14)	-0.07 (-0.21, 0.07)	-0.11 (-0.25, 0.03)
Age			
20-40 years old			
FT3 (pg/mL)	1.51 (0.82, 2.19) ***	0.39 (-0.32, 1.11)	0.19 (-0.54, 0.91)
FT4 (ng/dL)	0.44 (-1.84, 2.72)	-0.84 (-3.09, 1.40)	-0.78 (-3.02, 1.46)
TSH (mIU/L)	0.09 (-0.20, 0.38)	0.11 (-0.17, 0.40)	0.00 (-0.29, 0.29)
TT3 (ng/dL)	0.00 (-0.01, 0.02)	-0.00 (-0.01, 0.01)	-0.01 (-0.02, 0.01)
TT4 (ug/dL)	-0.03 (-0.21, 0.14)	0.01 (-0.16, 0.18)	-0.03 (-0.20, 0.15)
40-60 years old			
FT3 (pg/mL)	1.78 (1.09, 2.47) ***	1.15 (0.44, 1.86) **	1.09 (0.31, 1.87) **
FT4 (ng/dL)	-1.05 (-3.18, 1.08)	-1.30 (-3.39, 0.78)	-1.26 (-3.42, 0.89)
TSH (mIU/L)	0.00 (-0.25, 0.26)	0.13 (-0.12, 0.38)	0.08 (-0.18, 0.34)
TT3 (ng/dL)	0.00 (-0.01, 0.01)	-0.00 (-0.01, 0.01)	-0.00 (-0.02, 0.01)
TT4 (ug/dL)	-0.10 (-0.26, 0.07)	-0.07 (-0.23, 0.10)	-0.09 (-0.26, 0.08)
60-80 years old			
FT3 (pg/mL)	1.69 (0.89, 2.48) ***	1.47 (0.66, 2.27) ***	1.31 (0.47, 2.16) **
FT4 (ng/dL)	-1.48 (-3.46, 0.50)	-1.29 (-3.27, 0.69)	-1.18 (-3.21, 0.85)
TSH (mIU/L)	-0.13 (-0.37, 0.12)	-0.15 (-0.40, 0.10)	-0.15 (-0.41, 0.10)
TT3 (ng/dL)	0.02 (0.01, 0.03) **	0.02 (0.00, 0.03) **	0.01 (0.00, 0.03) *
TT4 (ug/dL)	-0.15 (-0.32, 0.02)	-0.16 (-0.33, 0.01)	-0.16 (-0.33, 0.02)
BMI			
<25 kg/m^2^			
FT3 (pg/mL)	1.52 (0.84, 2.20) ***	1.13 (0.37, 1.89) **	0.94 (0.15, 1.73) *
FT4 (ng/dL)	-2.34 (-4.44, -0.25) *	-2.76 (-4.84, -0.68) *	-2.91 (-5.03, -0.79) **
TSH (mIU/L)	-0.07 (-0.33, 0.19)	-0.06 (-0.32, 0.21)	-0.07 (-0.34, 0.20)
TT3 (ng/dL)	0.01 (-0.01, 0.02)	0.00 (-0.01, 0.02)	0.00 (-0.01, 0.01)
TT4 (ug/dL)	-0.15 (-0.32, 0.01)	-0.14 (-0.30, 0.03)	-0.12 (-0.29, 0.04)
25-30 kg/m^2^			
FT3 (pg/mL)	1.27 (0.66, 1.88) ***	0.73 (0.06, 1.41) *	0.64 (-0.08, 1.37)
FT4 (ng/dL)	0.55 (-1.51, 2.61)	0.10 (-1.94, 2.15)	-0.01 (-2.08, 2.06)
TSH (mIU/L)	-0.22 (-0.48, 0.04)	-0.14 (-0.40, 0.12)	-0.10 (-0.37, 0.16)
TT3 (ng/dL)	-0.00 (-0.01, 0.01)	-0.00 (-0.02, 0.01)	-0.01 (-0.02, 0.00)
TT4 (ug/dL)	-0.20 (-0.37, -0.04) *	-0.19 (-0.36, -0.02) *	-0.17 (-0.34, 0.00)
≥30 kg/m^2^			
FT3 (pg/mL)	1.64 (0.93, 2.36) ***	0.82 (0.04, 1.61) *	0.82 (0.01, 1.63) *
FT4 (ng/dL)	-0.63 (-2.83, 1.58)	-0.49 (-2.67, 1.69)	-0.01 (-2.23, 2.22)
TSH (mIU/L)	0.03 (-0.24, 0.30)	0.12 (-0.15, 0.39)	0.10 (-0.17, 0.37)
TT3 (ng/dL)	0.01 (0.00, 0.03) *	0.01 (-0.01, 0.02)	0.01 (-0.01, 0.02)
TT4 (ug/dL)	-0.07 (-0.25, 0.10)	-0.03 (-0.21, 0.15)	-0.00 (-0.19, 0.18)

*P<0.05; **P<0.01; ***P<0.001.

FT3, free triiodothyronine; FT4, free thyroxine; TSH, thyroid stimulating hormone; TT3, total triiodothyronine; TT4, total thyroxine; BMI, body mass index.

Model 2: Adjusted for age, gender and race. Model 3: Adjusted for age, gender, race, education, marriages, body mass index, alcohol use, smoking status, total calcium, energy intake, Patient Health Questionnaire score, hypertension, diabetes and physical activities. All the models are not adjusted for the variable itself in each stratification.

By analyzing the subclinical hypothyroid and subclinical hyperthyroid populations separately, no statistical differences were observed in bowel habits (**Supplementary Tables 4, 5**). After comparing the gender differences in bowel habits between these two populations, we found that in participants with subclinical hyperthyroidism, the prevalence of chronic constipation was higher in women than in men (20.36% *vs.* 0.95%), while the prevalence of chronic diarrhea was lower than in men (2.86% *vs.* 10.87%). The differences were not statistically significant in patients with subclinical hypothyroidism (**Supplementary Tables 6, 7**). By analyzing the patients with and without subclinical hypothyroid, no statistical differences were observed in bowel habits (**Supplementary Table 8**).

### Smooth curve fitting and threshold effect analysis of the associations of thyroid hormone (FT3, FT4) concentrations with the risk of developing chronic diarrhea and the number of bowel movements

**Figure 2:**
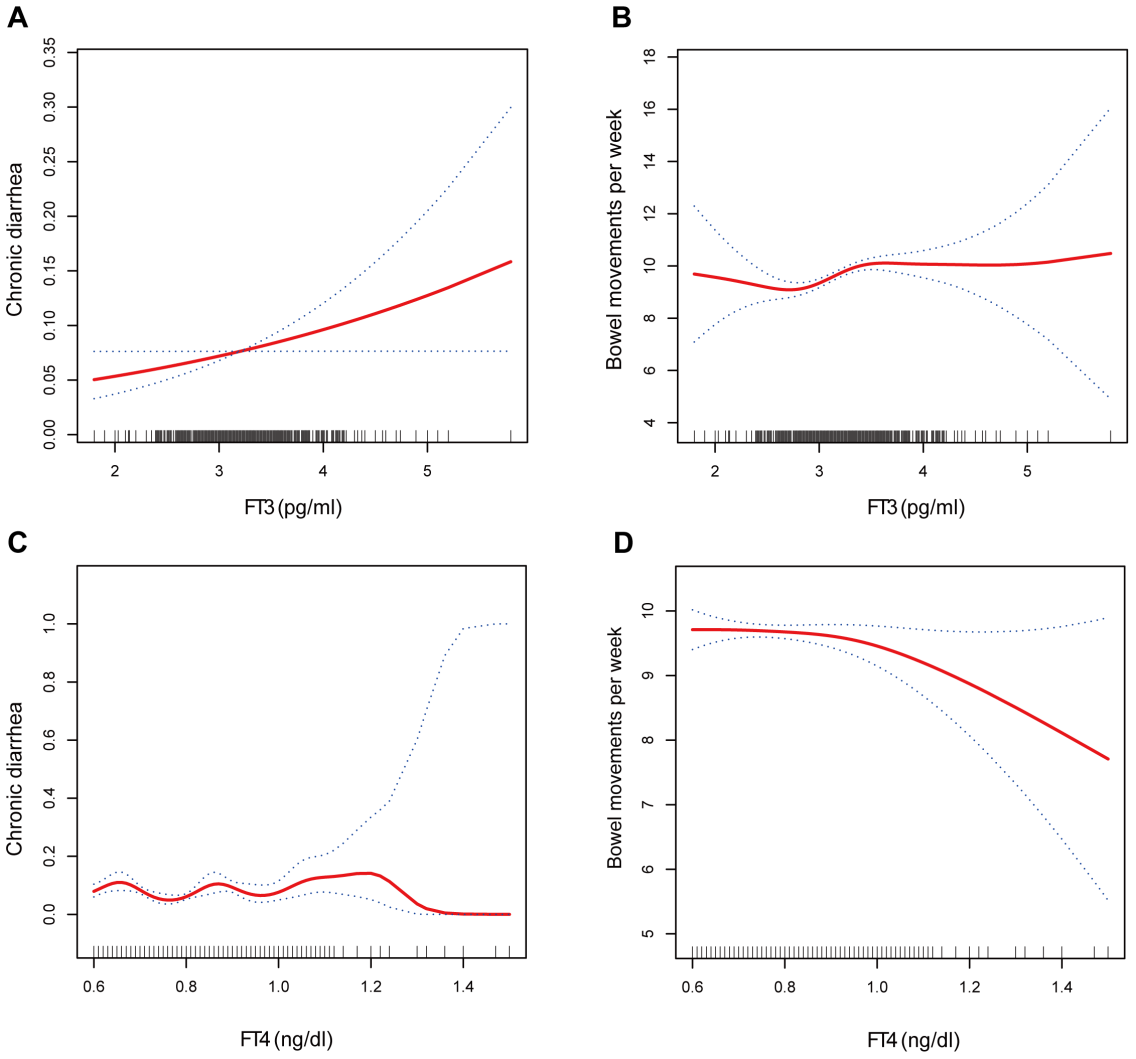
The association between **(A)** FT3 and chronic diarrhea, **(B)** FT3 and bowel movements, **(C)** FT4 and chronic diarrhea, and **(D)** FT4 and bowel movements.

The associations of the levels of thyroid hormones (FT3 and FT4) with the risk of developing chronic diarrhea and the number of bowel movements were then described via smoothed curve fitting (**Figure 2**). Smooth curve fitting revealed nonlinear relationships between FT4 concentrations and the risk of developing chronic diarrhea (**Figure 2C**). In addition, a two-stage regression model was employed to estimate the threshold effect (**Tables 5**, **6**). For the association between FT4 concentrations and chronic diarrhea, we found an inflection point of 0.79 ng/dl. Below the inflection point, FT4 concentrations were not correlated with the risk of developing chronic diarrhea (OR=0.22, 95% CI: 0.04-1.34), but above the inflection point, there was a positive correlation (OR=5.33, 95% CI: 1.40-20.40).

**Table 5:** Threshold effect analysis of FT3 on chronic diarrhea and bowel movements using two-piecewise linear regression model.

FT3	Adjusted OR/β (95% CI) *P* value	
	Chronic diarrhea	Bowel movements numbers
Inflection point	3.29	3.49
< Inflection point	1.16 (0.69, 1.97) 0.577	1.24 (0.63, 1.85) <0.001
> Inflection point	1.62 (0.96, 2.74) 0.071	-0.07 (-1.10, 0.97) 0.901
Log likelihood ratio	0.448	0.058

FT3, free triiodothyronine.

Age, gender, race, education, marriages, body mass index, alcohol use, smoking status, total calcium, energy intake, Patient Health Questionnaire score, hypertension, diabetes and physical activities were adjusted.

**Table 6:** Threshold effect analysis of FT4 on chronic diarrhea and bowel movements using two-piecewise linear regression model.

FT4	Adjusted OR/β (95% CI) *P* value	
	Chronic diarrhea	Bowel movements numbers
Inflection point	0.79	0.99
< Inflection point	0.22 (0.04, 1.34) 0.099	0.03 (-1.42, 1.48) 0.967
> Inflection point	5.33 (1.40, 20.40) 0.014	-6.87 (-12.30, -1.44) 0.013
Log likelihood ratio	0.021	0.025

FT4, free thyroxine.

Age, gender, race, education, marriages, body mass index, alcohol use, smoking status, total calcium, energy intake, Patient Health Questionnaire score, hypertension, diabetes and physical activities were adjusted.

## Discussion

With this population-based study from the NHANES, we discovered associations between thyroid hormone levels and abnormal bowel habits, especially between FT3 levels and the risk of developing chronic diarrhea. An increased risk of developing chronic diarrhea and an increased frequency of bowel movements are associated with elevated FT3 levels, even after controlling for confounding factors. Additionally, different gender, age, and BMI groups had different relationships with FT3, chronic diarrhea risk and the number of bowel movements.

To date, there have been few reports on the associations between abnormal bowel habits and thyroid hormones in euthyroid individuals. Thyroid dysfunction may be linked to and/or aggravate underlying gastrointestinal disease, or it may manifest as gastrointestinal symptoms. Similarly, since the intestinal tract contains a series of important thyroid hormone transporters, receptors, and deiodinase enzymes, intestinal dysfunction may also affect the absorption of thyroid hormones, such as T3 (19). The most prevalent digestive symptoms of hypothyroidism are constipation and a decreased frequency of stools (13). In hypothyroid rats, a reduction in daily fecal output, dilatation of the colon, and a reduced frequency of rhythmic colonic movement were observed (20). It is well known that hyperthyroidism can cause intestinal problems. One-quarter of individuals with hyperthyroidism develop mild to moderate diarrhea and experience an increased number of bowel movements (21). In one study, 7 patients with hyperthyroidism had more frequent bowel movements, whereas 3 out of 10 patients experienced diarrhea (22). According to a previous study, individuals with Grave’s disease had a fivefold increased risk of developing celiac disease compared with controls (23). These findings suggest that the intestinal hypermotility caused by hyperthyroidism shortens the intestinal transit time and causes diarrhea. Additionally, diarrhea is also caused by excessive intestinal mucosal secretion or elevated beta-adrenergic activity in hyperthyroid states (24), implying that the adrenergic/catecholamine system may play a role in dysmotility. Notably, average anal resting and squeezing pressures as well as the rectal threshold of sensitivity are lower in hyperthyroidism with diarrhea than in controls. While under hypothyroid accompanied by constipation conditions, the anorectal physiology is also disturbed. The maximum tolerable volume is lower and the threshold for rectal perception is greater than that of controls (25). Since patients with constipation or IBS may have alterations in visceral sensitivity, it is difficult to explain this alteration in bowel function by thyroid signaling dysregulation alone. However, this partially implied a relationship between thyroid function and visceral sensitivity. Our study revealed that diarrhea status was more strongly associated with FT3 concentrations in euthyroid individuals, which may be due to the stronger activity or sensitivity of FT3. Complex physiological changes in the HPT axis also develop with age (26, 27). Therefore, we speculate that the correlations between thyroid function and the number of bowel movements may not be as significant in young people as it is in middle-aged or older adults because of their greater sensitivity to negative thyroid hormone feedback or TSH activity and more active compensatory mechanisms. Similarly, the correlation varies across different genders or different BMI populations for other reasons, such as the influence of sex hormones (28). A significant negative correlation between FT3 concentrations and chronic constipation was observed in male participants aged 60–80 years. Thus, FT3 concentrations may partly explain the lower incidence of chronic constipation in male participants than that in females. Interestingly, we also found that in men, the risk of developing chronic diarrhea was positively associated with FT3 concentrations, whereas in women, the risk of developing chronic diarrhea was positively associated with FT4 concentrations. Further research is needed in the future.

The precise mechanism behind the association between thyroid hormone concentrations and abnormal bowel habits remains unclear. Research has revealed that intestinal dysmotility may be mediated through the inhibition of Cl^-^/HCO3^-^ anion exchange, which may result from T4 deficiency (29). T3 controls the balance of proliferation and differentiation of intestinal epithelial cells, mainly through interaction with TRα1. Compared with wild-type mice, TRα knockout mice presented reduced digestive enzyme levels, diminished intestinal function, and abnormal development of the intestine (30), indicating an essential role for TH in the maintenance of normal gastrointestinal function. Bao L et al. reported severe constipation in a transgenic mouse model with a dominant-negative mutation in TR, a model in which the interaction of T3 with TRs is blocked (31). Furthermore, symptoms of gastrointestinal dysfunction are present in approximately 80% of patients with dominant-negative mutations in TRα. Owing to dilated intestines, prolonged transit times, and impaired intestinal transit performance, the majority of these patients complain of various degrees of constipation (32). Thus, T3 may act by binding to receptors and may be involved in intestinal motility or secretory functions. More research is warranted to confirm the connection between thyroid hormone concentrations and gastrointestinal health, as well as to uncover the underlying mechanisms involved. In addition, one study revealed that the abundance of certain gut flora was associated with FT3 and FT4 concentrations in patients with hypothyroidism (33). Intestinal dysbiosis in primary hypothyroidism can reduce short-chain fatty acid (SCFA)-producing bacteria, leading to a disordered intestinal barrier in mice, which suggests that the gut flora may mediate thyroid-gut crosstalk (33). Furthermore, it has been reported that more than 1/2 of patients with hypothyroidism have the presence of small intestinal bacterial overgrowth (11), which may exacerbate bloating or intestinal motility disorders.

The advantages of our research are as follows. First, the large sample size from the NHANES improved the reliability and representativeness of our study. Furthermore, modifying confounding factors to better ensure that these findings are trustworthy and are suitable for use with the broader population. However, there are several limitations to this research. First, there was bound to be recall bias because the bowel habits were in accordance with personal interviews. Second, we categorized the population primarily by using the bowel health questionnaire, which asked participants about their “usual or most common” stool type. Therefore, we could not determine whether the participants fully satisfied the Rome criteria for functional constipation or diarrhea. Finally, because the study was cross-sectional in nature, we were unable to draw an exact causal link between bowel habits and thyroid hormone concentrations.

## Conclusion

In this study, we revealed the connection between thyroid hormone and bowel habits in euthyroid populations, particularly between FT3 concentrations and the risk of developing chronic diarrhea. A higher FT3 level was associated with an increased risk of developing chronic diarrhea and more frequent bowel movements. The associations between bowel habits and thyroid hormones differed across age, gender, and BMI subgroups. It is critical to acknowledge that the gastrointestinal tract and thyroid have complex interactions that affect both health and disease instead of functioning independently. To validate our results, further large-scale prospective studies are needed.

## Data Availability

Publicly available datasets were analyzed in this study. This data can be found here: www.cdc.gov/nchs/nhanes/.
