# Supplementary material for: Associations of thyroid hormones with chronic diarrhea and constipation in euthyroid individuals
Source: Front Endocrinol (Lausanne). 2024 Oct 17;15:1395743. doi: 10.3389/fendo.2024.1395743 (PMC11524803; doi:10.3389/fendo.2024.1395743)
Supplement: Supplementary file 1 [file DataSheet1.docx]

**Supplementary Table 1.** Thyroid hormone level and bowel health of euthyroid participants.

|  | Gender | | *P* value |
| --- | --- | --- | --- |
| **20-40 years old** | Male (n=998) | Female (n=847) |  |
| FT3 (pg/mL) | 3.46 ± 0.37 | 3.19 ± 0.34 | <0.001 |
| FT4 (ng/dL) | 0.80 ± 0.12 | 0.77 ± 0.11 | <0.001 |
| TSH (mIU/L) | 1.65 ± 0.92 | 1.64 ± 0.87 | 0.819 |
| TT3 (ng/dL) | 119.57 ± 19.65 | 118.18 ± 26.00 | 0.191 |
| TT4 (ug/dL) | 7.53 ± 1.38 | 8.01 ± 1.59 | <0.001 |
| Bowel health |  |  | <0.001 |
| Normal bowel habits (%) | 90.74 | 81.97 |  |
| Chronic constipation (%) | 4.94 | 11.04 |  |
| Chronic diarrhea (%) | 4.32 | 6.99 |  |
| **40-60 years old** | Male (n=930) | Female (n=794) |  |
| FT3 (pg/mL) | 3.27 ± 0.31 | 3.08 ± 0.35 | <0.001 |
| FT4 (ng/dL) | 0.77 ± 0.12 | 0.76 ± 0.12 | 0.054 |
| TSH (mIU/L) | 1.88 ± 1.02 | 1.89 ± 0.98 | 0.935 |
| TT3 (ng/dL) | 116.00 ± 19.89 | 112.41 ± 24.14 | <0.001 |
| TT4 (ug/dL) | 7.59 ± 1.34 | 7.89 ± 1.55 | <0.001 |
| Bowel health |  |  | <0.001 |
| Normal bowel habits (%) | 87.87 | 82.69 |  |
| Chronic constipation (%) | 5.08 | 10.02 |  |
| Chronic diarrhea (%) | 7.06 | 7.29 |  |
| **60-80 years old** | Male (n=756) | Female (n=674) |  |
| FT3 (pg/mL) | 3.06 ± 0.32 | 2.96 ± 0.30 | <0.001 |
| FT4 (ng/dL) | 0.80 ± 0.13 | 0.80 ± 0.13 | 0.438 |
| TSH (mIU/L) | 2.01 ± 1.02 | 2.07 ± 1.05 | 0.354 |
| TT3 (ng/dL) | 106.05 ± 19.95 | 104.96 ± 20.28 | 0.308 |
| TT4 (ug/dL) | 7.78 ± 1.48 | 7.97 ± 1.56 | 0.018 |
| Bowel health |  |  | <0.001 |
| Normal bowel habits (%) | 89.58 | 81.20 |  |
| Chronic constipation (%) | 2.48 | 10.06 |  |
| Chronic diarrhea (%) | 7.94 | 8.75 |  |

Mean ± SD for continuous variables. % for categorical variables.

FT3, free triiodothyronine; FT4, free thyroxine; TSH, thyroid stimulating hormone; TT3, total triiodothyronine; TT4, total thyroxine.

**Supplementary Table 2.** The associations of thyroid hormone with chronic constipation based on different ages.

| Exposure | OR (95% CI), *P* value | | |
| --- | --- | --- | --- |
|  | 20-40 years old | 40-60 years old | 60-80 years old |
| **Male** |  |  |  |
| Model 1 (Non-adjusted) |  |  |  |
| FT3 (pg/mL) | 1.70 (0.84, 3.47) | 1.05 (0.44, 2.51) | 0.23 (0.07, 0.75) * |
| FT4 (ng/dL) | 2.79 (0.29, 26.33) | 0.47 (0.04, 5.40) | 0.50 (0.03, 9.24) |
| TSH (mIU/L) | 1.07 (0.80, 1.44) | 1.02 (0.77, 1.36) | 0.88 (0.60, 1.29) |
| TT3 (ng/dL) | 1.01 (1.00, 1.03) | 1.01 (0.99, 1.02) | 1.00 (0.98, 1.01) |
| TT4 (ug/dL) | 1.03 (0.85, 1.24) | 1.06 (0.87, 1.30) | 1.03 (0.81, 1.31) |
| Model 2 (Adjust I) |  |  |  |
| FT3 (pg/mL) | 1.63 (0.79, 3.36) | 1.06 (0.43, 2.59) | 0.22 (0.07, 0.72) * |
| FT4 (ng/dL) | 2.59 (0.26, 25.67) | 0.52 (0.05, 5.93) | 0.56 (0.03, 10.99) |
| TSH (mIU/L) | 1.06 (0.79, 1.43) | 1.04 (0.78, 1.38) | 0.86 (0.58, 1.28) |
| TT3 (ng/dL) | 1.01 (1.00, 1.02) | 1.01 (0.99, 1.02) | 0.99 (0.98, 1.01) |
| TT4 (ug/dL) | 1.01 (0.83, 1.23) | 1.07 (0.87, 1.30) | 1.04 (0.81, 1.34) |
| Model 3 (Adjust II) |  |  |  |
| FT3 (pg/mL) | 1.66 (0.79, 3.48) | 0.78 (0.29, 2.09) | 0.19 (0.05, 0.80) * |
| FT4 (ng/dL) | 2.29 (0.21, 24.49) | 0.38 (0.03, 5.18) | 0.42 (0.02, 10.95) |
| TSH (mIU/L) | 1.00 (0.73, 1.38) | 0.99 (0.73, 1.33) | 0.83 (0.54, 1.28) |
| TT3 (ng/dL) | 1.01 (1.00, 1.03) | 1.00 (0.99, 1.02) | 0.99 (0.97, 1.02) |
| TT4 (ug/dL) | 1.01 (0.83, 1.22) | 1.04 (0.84, 1.27) | 0.97 (0.72, 1.31) |
| **Female** |  |  |  |
| Model 1 (Non-adjusted) |  |  |  |
| FT3 (pg/mL) | 1.52 (0.86, 2.69) | 0.49 (0.24, 1.03) | 1.92 (0.81, 4.56) |
| FT4 (ng/dL) | 5.39 (0.82, 35.33) | 0.20 (0.02, 1.63) | 0.22 (0.02, 2.13) |
| TSH (mIU/L) | 0.94 (0.73, 1.19) | 0.69 (0.52, 0.91) ** | 1.10 (0.87, 1.40) |
| TT3 (ng/dL) | 1.00 (1.00, 1.01) | 1.00 (0.99, 1.01) | 1.00 (0.99, 1.02) |
| TT4 (ug/dL) | 0.98 (0.86, 1.13) | 0.98 (0.85, 1.14) | 0.95 (0.79, 1.14) |
| Model 2 (Adjust I) |  |  |  |
| FT3 (pg/mL) | 1.56 (0.87, 2.79) | 0.50 (0.24, 1.05) | 1.96 (0.81, 4.74) |
| FT4 (ng/dL) | 5.04 (0.76, 33.63) | 0.23 (0.03, 1.83) | 0.18 (0.02, 1.78) |
| TSH (mIU/L) | 0.96 (0.75, 1.22) | 0.70 (0.53, 0.93) * | 1.06 (0.83, 1.36) |
| TT3 (ng/dL) | 1.00 (1.00, 1.01) | 1.00 (0.99, 1.01) | 1.01 (0.99, 1.02) |
| TT4 (ug/dL) | 0.98 (0.85, 1.12) | 0.99 (0.85, 1.14) | 0.97 (0.81, 1.17) |
| Model 3 (Adjust II) |  |  |  |
| FT3 (pg/mL) | 1.41 (0.77, 2.61) | 0.55 (0.26, 1.19) | 1.83 (0.67, 4.99) |
| FT4 (ng/dL) | 4.44 (0.61, 32.13) | 0.16 (0.02, 1.49) | 0.34 (0.03, 3.71) |
| TSH (mIU/L) | 1.02 (0.79, 1.31) | 0.70 (0.52, 0.94) * | 1.04 (0.79, 1.36) |
| TT3 (ng/dL) | 1.00 (1.00, 1.01) | 1.00 (0.99, 1.01) | 1.01 (0.99, 1.02) |
| TT4 (ug/dL) | 0.97 (0.84, 1.12) | 0.99 (0.85, 1.16) | 1.03 (0.85, 1.24) |
| **Total** |  |  |  |
| Model 1 (Non-adjusted) |  |  |  |
| FT3 (pg/mL) | 1.59 (1.02, 2.48) * | 0.67 (0.38, 1.16) | 0.90 (0.45, 1.79) |
| FT4 (ng/dL) | 4.09 (0.97, 17.22) | 0.29 (0.06, 1.41) | 0.30 (0.05, 1.79) |
| TSH (mIU/L) | 0.99 (0.82, 1.19) | 0.82 (0.67, 1.00) | 1.03 (0.84, 1.26) |
| TT3 (ng/dL) | 1.01 (1.00, 1.01) | 1.00 (0.99, 1.01) | 1.00 (0.99, 1.01) |
| TT4 (ug/dL) | 1.00 (0.89, 1.12) | 1.01 (0.90, 1.13) | 0.98 (0.85, 1.13) |
| Model 2 (Adjust I) |  |  |  |
| FT3 (pg/mL) | 1.59 (1.01, 2.49) * | 0.67 (0.39, 1.17) | 0.88 (0.44, 1.78) |
| FT4 (ng/dL) | 4.15 (0.97, 17.66) | 0.32 (0.07, 1.58) | 0.27 (0.04, 1.64) |
| TSH (mIU/L) | 1.00 (0.83, 1.21) | 0.83 (0.68, 1.02) | 1.00 (0.81, 1.23) |
| TT3 (ng/dL) | 1.01 (1.00, 1.01) | 1.00 (0.99, 1.01) | 1.00 (0.99, 1.01) |
| TT4 (ug/dL) | 0.99 (0.88, 1.11) | 1.01 (0.90, 1.14) | 0.98 (0.85, 1.14) |
| Model 3 (Adjust II) |  |  |  |
| FT3 (pg/mL) | 1.57 (0.98, 2.49) | 0.62 (0.34, 1.12) | 0.75 (0.34, 1.65) |
| FT4 (ng/dL) | 3.95 (0.90, 17.27) | 0.24 (0.04, 1.26) | 0.37 (0.06, 2.37) |
| TSH (mIU/L) | 1.01 (0.83, 1.23) | 0.83 (0.68, 1.02) | 1.00 (0.80, 1.25) |
| TT3 (ng/dL) | 1.01 (1.00, 1.01) | 1.00 (0.99, 1.01) | 1.00 (0.99, 1.01) |
| TT4 (ug/dL) | 0.99 (0.88, 1.10) | 1.01 (0.89, 1.14) | 1.00 (0.86, 1.17) |

**P*<0.05; ***P*<0.01; ****P*<0.001

FT3, free triiodothyronine; FT4, free thyroxine; TSH, thyroid stimulating hormone; TT3, total triiodothyronine; TT4, total thyroxine.

Model 2: Adjusted for race. Model 3: Adjusted for race, education, marriages, body mass index, alcohol use, smoking status, total calcium, energy intake, Patient Health Questionnaire score, hypertension, diabetes and physical activities.

**Supplementary Table 3.** The associations of thyroid hormone with chronic diarrhea based on different ages.

| Exposure | OR (95% CI), *P* value | | |
| --- | --- | --- | --- |
|  | 20-40 years old | 40-60 years old | 60-80 years old |
| **Male** |  |  |  |
| Model 1 (Non-adjusted) |  |  |  |
| FT3 (pg/mL) | 2.40 (1.14, 5.02) * | 1.51 (0.71, 3.24) | 1.28 (0.60, 2.72) |
| FT4 (ng/dL) | 3.61 (0.33, 39.88) | 0.35 (0.04, 3.11) | 0.55 (0.08, 3.92) |
| TSH (mIU/L) | 1.02 (0.74, 1.42) | 0.96 (0.74, 1.25) | 0.97 (0.76, 1.25) |
| TT3 (ng/dL) | 1.01 (1.00, 1.03) | 1.01 (1.00, 1.02) | 1.00 (0.99, 1.02) |
| TT4 (ug/dL) | 1.21 (1.03, 1.43) * | 0.96 (0.80, 1.15) | 1.00 (0.84, 1.18) |
| Model 2 (Adjust I) |  |  |  |
| FT3 (pg/mL) | 2.30 (1.08, 4.88) * | 1.51 (0.70, 3.28) | 1.21 (0.56, 2.58) |
| FT4 (ng/dL) | 3.02 (0.26, 35.43) | 0.34 (0.04, 3.09) | 0.54 (0.07, 3.88) |
| TSH (mIU/L) | 1.00 (0.72, 1.39) | 0.98 (0.75, 1.28) | 0.96 (0.74, 1.24) |
| TT3 (ng/dL) | 1.01 (1.00, 1.03) | 1.01 (1.00, 1.02) | 1.00 (0.99, 1.02) |
| TT4 (ug/dL) | 1.22 (1.03, 1.45) * | 0.95 (0.79, 1.15) | 0.99 (0.84, 1.18) |
| Model 3 (Adjust II) |  |  |  |
| FT3 (pg/mL) | 2.40 (1.10, 5.26) * | 1.89 (0.84, 4.27) | 1.42 (0.61, 3.31) |
| FT4 (ng/dL) | 2.50 (0.20, 31.58) | 0.30 (0.03, 2.99) | 0.29 (0.03, 2.60) |
| TSH (mIU/L) | 0.94 (0.67, 1.33) | 0.92 (0.70, 1.22) | 0.92 (0.69, 1.21) |
| TT3 (ng/dL) | 1.01 (1.00, 1.03) | 1.02 (1.00, 1.03) * | 1.01 (0.99, 1.02) |
| TT4 (ug/dL) | 1.22 (1.01, 1.46) * | 0.95 (0.78, 1.15) | 1.00 (0.83, 1.21) |
| **Female** |  |  |  |
| Model 1 (Non-adjusted) |  |  |  |
| FT3 (pg/mL) | 1.29 (0.63, 2.65) | 1.69 (0.99, 2.88) | 1.34 (0.63, 2.87) |
| FT4 (ng/dL) | 0.27 (0.02, 3.26) | 21.94 (3.02, 159.29) ** | 2.90 (0.49, 17.23) |
| TSH (mIU/L) | 1.20 (0.92, 1.57) | 0.96 (0.74, 1.23) | 0.90 (0.72, 1.14) |
| TT3 (ng/dL) | 1.00 (0.98, 1.01) | 1.00 (0.99, 1.01) | 1.01 (1.00, 1.02) |
| TT4 (ug/dL) | 0.87 (0.73, 1.04) | 1.11 (0.97, 1.28) | 1.06 (0.91, 1.23) |
| Model 2 (Adjust I) |  |  |  |
| FT3 (pg/mL) | 1.26 (0.61, 2.58) | 1.54 (0.89, 2.65) | 1.31 (0.61, 2.82) |
| FT4 (ng/dL) | 0.29 (0.02, 3.48) | 22.26 (3.04, 163.21) ** | 3.70 (0.62, 22.09) |
| TSH (mIU/L) | 1.19 (0.91, 1.55) | 0.98 (0.76, 1.26) | 0.95 (0.75, 1.20) |
| TT3 (ng/dL) | 0.99 (0.98, 1.01) | 1.00 (0.99, 1.01) | 1.01 (1.00, 1.02) |
| TT4 (ug/dL) | 0.86 (0.72, 1.04) | 1.09 (0.95, 1.26) | 1.03 (0.88, 1.20) |
| Model 3 (Adjust II) |  |  |  |
| FT3 (pg/mL) | 1.00 (0.47, 2.13) | 1.06 (0.48, 2.33) | 1.32 (0.57, 3.03) |
| FT4 (ng/dL) | 0.20 (0.02, 2.63) | 78.01 (8.67, 701.57) *** | 3.13 (0.47, 20.69) |
| TSH (mIU/L) | 1.12 (0.85, 1.48) | 1.00 (0.76, 1.31) | 0.97 (0.75, 1.24) |
| TT3 (ng/dL) | 0.99 (0.98, 1.00) | 0.99 (0.98, 1.00) | 1.01 (1.00, 1.02) |
| TT4 (ug/dL) | 0.84 (0.69, 1.02) | 1.12 (0.96, 1.30) | 1.01 (0.86, 1.20) |
| **Total** |  |  |  |
| Model 1 (Non-adjusted) |  |  |  |
| FT3 (pg/mL) | 1.72 (1.03, 2.87) * | 1.63 (1.06, 2.52) * | 1.31 (0.77, 2.24) |
| FT4 (ng/dL) | 0.97 (0.17, 5.56) | 3.03 (0.71, 12.86) | 1.33 (0.36, 4.88) |
| TSH (mIU/L) | 1.12 (0.92, 1.38) | 0.96 (0.80, 1.15) | 0.93 (0.79, 1.11) |
| TT3 (ng/dL) | 1.00 (0.99, 1.01) | 1.00 (1.00, 1.01) | 1.01 (1.00, 1.01) |
| TT4 (ug/dL) | 1.02 (0.89, 1.16) | 1.05 (0.94, 1.18) | 1.03 (0.92, 1.15) |
| Model 2 (Adjust I) |  |  |  |
| FT3 (pg/mL) | 1.67 (1.00, 2.80) | 1.54 (0.99, 2.39) | 1.23 (0.72, 2.12) |
| FT4 (ng/dL) | 0.93 (0.16, 5.42) | 3.08 (0.72, 13.15) | 1.52 (0.41, 5.64) |
| TSH (mIU/L) | 1.11 (0.90, 1.36) | 0.98 (0.81, 1.17) | 0.95 (0.80, 1.13) |
| TT3 (ng/dL) | 1.00 (0.99, 1.01) | 1.00 (1.00, 1.01) | 1.00 (1.00, 1.01) |
| TT4 (ug/dL) | 1.01 (0.88, 1.15) | 1.04 (0.93, 1.17) | 1.02 (0.91, 1.14) |
| Model 3 (Adjust II) |  |  |  |
| FT3 (pg/mL) | 1.48 (0.87, 2.51) | 1.31 (0.75, 2.27) | 1.37 (0.77, 2.45) |
| FT4 (ng/dL) | 0.78 (0.13, 4.70) | 3.87 (0.85, 17.62) | 1.13 (0.29, 4.39) |
| TSH (mIU/L) | 1.04 (0.84, 1.29) | 0.96 (0.80, 1.17) | 0.95 (0.79, 1.13) |
| TT3 (ng/dL) | 1.00 (0.99, 1.01) | 1.00 (0.99, 1.01) | 1.01 (1.00, 1.02) |
| TT4 (ug/dL) | 0.99 (0.86, 1.13) | 1.04 (0.92, 1.17) | 1.01 (0.90, 1.15) |

**P*<0.05; ***P*<0.01; ****P*<0.001

FT3, free triiodothyronine; FT4, free thyroxine; TSH, thyroid stimulating hormone; TT3, total triiodothyronine; TT4, total thyroxine.

Model 2: Adjusted for race. Model 3: Adjusted for race, education, marriages, body mass index, alcohol use, smoking status, total calcium, energy intake, Patient Health Questionnaire score, hypertension, diabetes and physical activities.

**Supplementary Table 4.** Thyroid hormone level of subclinical hypothyroidism participants based on bowel habits.

|  | Normal bowel habits (n=79) | Chronic constipation (n=4) | Chronic diarrhea(n=14) | *P* value |
| --- | --- | --- | --- | --- |
| FT3 (pg/mL) | 3.13 ± 0.36 | 2.92 ± 0.28 | 3.17 ± 0.33 | 0.412 |
| FT4 (ng/dL) | 0.71 ± 0.09 | 0.79 ± 0.06 | 0.72 ± 0.12 | 0.254 |
| TSH (mIU/L) | 7.71 ± 2.88 | 6.49 ± 0.93 | 9.95 ± 7.31 | 0.116 |
| TT3 (ng/dL) | 114.03 ± 27.70 | 86.23 ± 11.07 | 118.36 ± 25.50 | 0.075 |
| TT4 (ug/dL) | 7.28 ± 1.45 | 7.92 ± 0.80 | 7.06 ± 1.42 | 0.547 |

Mean ± SD for continuous variables: the *P* value was calculated by the weighted linear regression model.

FT3, free triiodothyronine; FT4, free thyroxine; TSH, thyroid stimulating hormone; TT3, total triiodothyronine; TT4, total thyroxine.

**Supplementary Table 5.** Thyroid hormone level of subclinical hyperthyroidism participants based on bowel habits.

|  | Normal bowel habits (n=50) | Chronic constipation (n=8) | Chronic diarrhea(n=5) | *P* value |
| --- | --- | --- | --- | --- |
| FT3 (pg/mL) | 3.27 ± 0.41 | 3.32 ± 0.25 | 3.14 ± 0.16 | 0.755 |
| FT4 (ng/dL) | 0.90 ± 0.15 | 0.89 ± 0.26 | 1.10 ± 0.34 | 0.135 |
| TSH (mIU/L) | 0.20 ± 0.11 | 0.21 ± 0.09 | 0.30 ± 0.07 | 0.191 |
| TT3 (ng/dL) | 116.68 ± 27.29 | 117.20 ± 18.46 | 96.25 ± 20.85 | 0.330 |
| TT4 (ug/dL) | 8.73 ± 1.93 | 8.59 ± 2.09 | 9.60 ± 1.87 | 0.679 |

Mean ± SD for continuous variables: the *P* value was calculated by the weighted linear regression model.

FT3, free triiodothyronine; FT4, free thyroxine; TSH, thyroid stimulating hormone; TT3, total triiodothyronine; TT4, total thyroxine.

**Supplementary Table 6.** Bowel health of subclinical hypothyroidism participants based on gender.

|  | Male (n=50) | Female (n=47) | *P* value |
| --- | --- | --- | --- |
| Bowel health (%) |  |  | 0.2353 |
| Normal bowel habits | 89.48 | 77.02 |  |
| Chronic constipation | 2.50 | 7.94 |  |
| Chronic diarrhea | 8.02 | 15.04 |  |

**Supplementary Table 7.** Bowel health of subclinical hyperthyroidism participants based on gender.

|  | Male (n=30) | Female (n=33) | *P* value |
| --- | --- | --- | --- |
| Bowel health (%) |  |  | 0.0320 |
| Normal bowel habits | 88.18 | 76.78 |  |
| Chronic constipation | 0.95 | 20.36 |  |
| Chronic diarrhea | 10.87 | 2.86 |  |

**Supplementary Table 8.** Bowel health of participants with or without subclinical hypothyroidism.

|  | With subclinical hypothyroid (n=97) | Without subclinical hypothyroid  (n=5519) | *P* value |
| --- | --- | --- | --- |
| Bowel health (%) |  |  | 0.1839 |
| Normal bowel habits | 83.44 | 85.85 |  |
| Chronic constipation | 5.13 | 7.23 |  |
| Chronic diarrhea | 11.42 | 6.92 |  |
